# Effects of digging by a native and introduced ecosystem engineer on soil physical and chemical properties in temperate grassy woodland

**DOI:** 10.7717/peerj.7506

**Published:** 2019-08-20

**Authors:** Catherine E. Ross, Nicola T. Munro, Philip S. Barton, Maldwyn J. Evans, John Gillen, Ben C.T. Macdonald, Sue McIntyre, Saul A. Cunningham, Adrian D. Manning

**Affiliations:** 1 Fenner School of Environment and Society, Australian National University, Acton, ACT, Australia; 2 CSIRO, Black Mountain, ACT, Australia

**Keywords:** Eastern bettong, European rabbit, Ecosystem engineer, Soil nutrients, Grassland, Grassy woodland, Digging

## Abstract

Temperate grasslands and woodlands are the focus of extensive restoration efforts worldwide. Reintroduction of locally extinct soil-foraging and burrowing animals has been suggested as a means to restore soil function in these ecosystems. Yet little is known about the physical and chemical effects of digging on soil over time and how these effects differ between species of digging animal, vegetation types or ecosystems. We compared foraging pits of a native reintroduced marsupial, the eastern bettong (*Bettongia gaimardi*) and that of the exotic European rabbit (*Oryctolagus cuniculus*). We simulated pits of these animals and measured pit dimensions and soil chemical properties over a period of 2 years. We showed that bettong and rabbit pits differed in their morphology and longevity, and that pits had a strong moderating effect on soil surface temperatures. Over 75% of the simulated pits were still visible after 2 years, and bettong pits infilled faster than rabbit pits. Bettong pits reduced diurnal temperature range by up to 25 °C compared to the soil surface. We did not find any effects of digging on soil chemistry that were consistent across vegetation types, between bettong and rabbit pits, and with time since digging, which is contrary to studies conducted in arid biomes. Our findings show that animal foraging pits in temperate ecosystems cause physical alteration of the soil surface and microclimatic conditions rather than nutrient changes often observed in arid areas.

## Introduction

Temperate grasslands and woodlands are among the most threatened biomes worldwide due to widespread clearing and degradation from land use changes and inappropriate management (Hoekstra et al., 2004). Loss of species has both accompanied and contributed to this degradation, including soil-foraging and burrowing animals that play a role in soil turnover. Some of these animals are considered to be ‘ecosystem engineers’ because their digging behaviour has cascading effects on soil function and associated biota (Jones, Lawton & Shachak, 1994; Berke, 2010). Most knowledge of the role of digging animals in ecosystems has been developed from arid environments (Coggan, Hayward & Gibb, 2018). This leaves little understanding of their role in many other ecosystems, and their potential use for ecosystem restoration (Byers et al., 2006; Manning, Eldridge & Jones, 2015).

Ecosystem engineers structurally alter their environment, which in turn leads to changes in abiotic and biotic conditions (Jones et al., 2010). In the case of digging animals, the creation of pits and burrows can increase soil moisture and infiltration (Laundre, 1993; Garkaklis, Bradley & Wooller, 1998; Eldridge, 2009; Eldridge et al., 2012a; Valentine et al., 2017), reduce soil bulk density (Canals, Herman & Firestone, 2003; Cuevas et al., 2012; Travers et al., 2012), moderate extremes of temperature (Gutterman, 1997; Eldridge & Mensinga, 2007; James, Eldridge & Moseby, 2010), mix the soil profile and trap plant litter and seeds (Martin, 2003; Eldridge & Mensinga, 2007; James, Eldridge & Hill, 2009).

Several studies have found that digging animals can also change the chemistry of soils, but these effects are highly variable. Some nutrients (e.g. carbon, nitrogen, ammonium, nitrate, phosphorus and sulphur) may be higher in pits because of collection of organic matter (Tardiff & Stanford, 1998; James, Eldridge & Hill, 2009; Eldridge et al., 2012b; Travers et al., 2012), increased microbial activity and decomposition (Eldridge et al., 2015, 2016; Valentine et al., 2017), or removal of plants which would otherwise use the nutrients (Canals, Herman & Firestone, 2003). In contrast, some studies have found a reduction in certain nutrients in pits, perhaps as a result of leaching due to increased water infiltration (Garkaklis, Bradley & Wooller, 2003; Eldridge & Mensinga, 2007), and others have found no effect (Groot Bruinderink & Hazebroek, 1996). A recent global meta-analysis of the effects of digging animals on soil found they significantly increased soil N and P, but there was no overall effect for C or pH (Mallen-Cooper, Nakagawa & Eldridge, 2019).

Most studies of digging animals have focused on arid and semi-arid ecosystems (Whitford & Kay, 1999; Kinlaw, 1999), with few studies conducted in temperate ecosystems, particularly in Australia (Coggan, Hayward & Gibb, 2018). This is important because the effects of digging are likely to differ between arid and temperate ecosystems (Crain & Bertness, 2006). Several studies have suggested that the effects of digging animals are more pronounced in more arid or resource-poor sites (Mallen-Cooper, Nakagawa & Eldridge, 2019; Decker, Eldridge & Gibb, 2019), however Coggan, Hayward & Gibb (2016) found the opposite pattern. Further research on ecosystem engineers in temperate ecosystems is therefore required to close this knowledge gap.

The total impact of an ecosystem engineer on its environment depends on both the spatial and temporal aspects of its effects (Hastings et al., 2007). However, most studies on digging animals have focused on only the spatial aspects of digging; quantifying the number and distribution of pits and how much soil is moved in a certain area (e.g. Eldridge, 2004). In contrast, fewer studies have looked at temporal aspects of digging such as the longevity or ‘decay rate’ of pits (Raynaud, Jones & Barot, 2013). How long the effects of pits persist is likely to be influenced by many factors such as vegetation, soil type, climate and topography, as well as the morphology of the pit itself (Alkon, 1999). For example, pits have been shown to infill more quickly in sandy soils (Newell, 2008) and under tree canopies (Eldridge & Kwok, 2008), while pits with larger openings collect more litter (James, Eldridge & Hill, 2009). The effects of digging on soil chemistry are also likely to change over time as organic matter accumulates and decomposes and may persist well after the physical pit is no longer visible.

In Australia, habitat loss and feral predators have caused widespread decline of many native soil-foraging and burrowing mammals (Burbidge & McKenzie, 1989), and their loss is thought to have contributed to the degradation of Australian ecosystems (Martin, 2003; Eldridge & James, 2009; Fleming et al., 2014). However, the introduced European rabbit (*Oryctolagus cuniculus*) has become widespread in most ecosystems, and it has been suggested that they could fill a similar niche (Read et al., 2008; James et al., 2011). This is because they are comparable in size and create small foraging pits similar to those of native digging animals (James et al., 2011). In their native range, rabbits are recognised as important ecosystem engineers, increasing habitat heterogeneity and plant diversity (Gálvez-Bravo et al., 2011). In Australia, however, they have become extremely abundant, leading to negative impacts on soils and native vegetation (Eldridge & Myers, 2001; Eldridge & Simpson, 2002; Eldridge & Koen, 2008) and competition with native animals (Short & Smith, 1994; Short, 1998; Johnson, 2006). Rabbits also create fewer pits than other native species, and their pits tend to be shallower (James & Eldridge, 2007; James et al., 2011; Munro et al., 2019), which may have an impact on their ecological effects. To date, no studies have directly compared the physical and chemical properties of rabbit foraging pits with a native marsupial in a temperate ecosystem.

In this study, we wanted to investigate the physical and chemical effects of foraging pits of an Australian native marsupial, the eastern bettong (*Bettongia gaimardi*) and those of the introduced European rabbit. To do this, we accurately re-created pits and measured their physical dimensions and soil properties over time. We also used real bettong diggings for measuring the microclimatic effects on temperature. We posed the following questions:

1. How do the physical dimensions of artificial bettong and rabbit pits change over time?
2. Do natural bettong pits influence soil surface temperature?
3. What is the effect of artificial bettong and rabbit digging on soil chemistry within and directly beneath the pits, and how does this change over time?

We hypothesised that differences in morphology of bettong and rabbit foraging pits would result in different rates of infill over time, and that natural bettong pits would have a more mesic microclimate with a smaller diurnal temperature range compared to the soil surface. We also predicted that soil collected from within and beneath bettong pits would be distinct from rabbit pits and control (non-pit) sites, and that any effects on soil chemistry would change over time.

By addressing these questions, our study provides some new insight into the role of digging mammals as ecosystem engineers in temperate ecosystems and informs the conservation and management of both native and exotic digging mammals.

## Materials and Methods

### Study area

We conducted our study within the Mulligan’s Flat-Goorooyarroo Woodland Experiment, which consists of two neighbouring nature reserves on the outskirts of Canberra, south-eastern Australia (Manning et al., 2011; Shorthouse et al., 2012). The two reserves contain important remnants of Yellow Box-Blakely’s Red Gum Grassy Woodland, which is listed as a critically endangered ecological community (Australian Government, 2006). In 2009, a 485 ha predator-proof sanctuary was established in Mulligan’s Flat reserve to provide protection for the native wildlife and allow the reintroduction of several locally extinct species, including the eastern bettong (*Bettongia gaimardi*) which was introduced in 2012 (Batson et al., 2016). Within the sanctuary, feral predators (cats, foxes and dogs) and hares were removed, and rabbits were managed at low numbers.

### Vegetation, soils and climate

The soils and vegetation in the reserve have been described by Lepschi (1993) and McIntyre et al. (2010). For this study we defined three structural vegetation types: ‘Grassland’ (dominated by *Rytidosperma sp.*, with poorer soils); ‘Woodland’ (discontinuous eucalyptus canopy with understorey of *Themeda australis* and large tussock grass e.g. *Rytidosperma pallida*, richer soils) and ‘Forest’ (continuous eucalyptus canopy with sparse understorey of *Rytidosperma pallida*, with intermediate soils and thick litter layer).

Mean minimum and maximum temperatures for the hottest and coldest month are 13 °C and 28 °C (Jan) and 0 °C and 11 °C (Jul) respectively. Mean annual rainfall is 644.5 mm (1935–2017, Bureau of Meteorology, 2018). Monthly rainfall over the study period is shown in Fig. S1 (see Supplementary Materials).

### Study design

We assessed the physical and chemical properties of artificial bettong and rabbit pits located in the three vegetation types and over time. Our study design consisted of three fenced bettong ‘exclosures’ (200 m × 50 m) within the reserve, with one in each of the three vegetation types. We used fenced areas where bettongs did not have access to prevent any subsequent disturbance. We marked transects with star pickets placed 50 m from one end of the exclosure extending through the middle of the site for 50 m. In the woodland site, the transect passed through a section of grassland indicating a potentially different soil type, so we extended the transect to 70 m and avoided taking soil samples from that section. In December 2014, we placed artificial pits (see below) one metre apart along the transect, alternating between bettong and rabbit pits (giving a total of 170 pits—85 bettongs and 85 rabbits). The location of each pit was marked with a peg and a metal tag. For each pit, we measured length, width and depth (at the deepest point). We placed three coloured pebbles (approx. five mm in diameter) in the bottom of each pit to mark the original depth. In August 2015 (8 months after initiation), the pits were measured again. We then took soil samples from a selection of the pits (see below). This was repeated in January 2017 (24 months after initiation).

### Artificial pits

**Figure 1:**
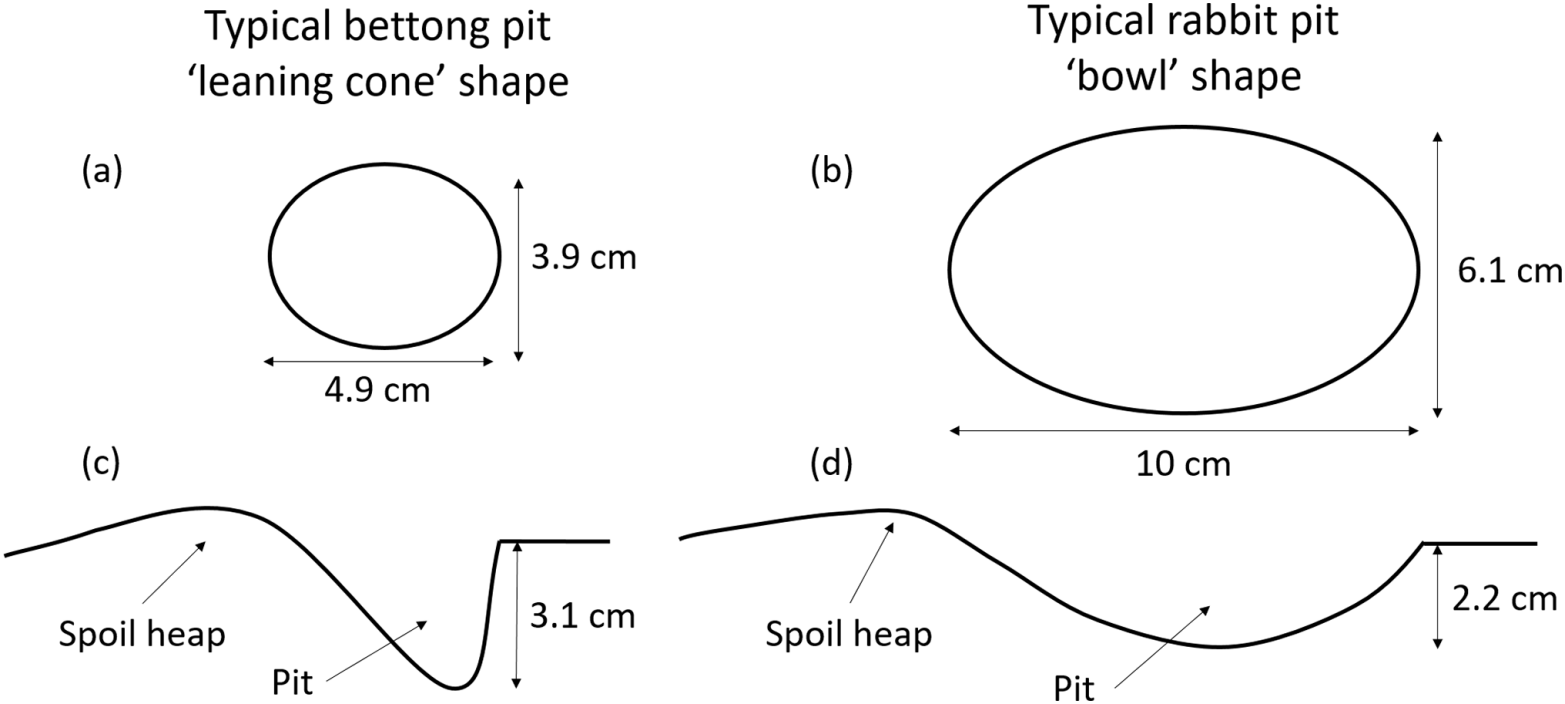
Bettong and rabbit pit dimensions. Shape and dimensions of ‘typical’ bettong and rabbit foraging pits shown from above (A, B) and in cross-section (C, D) (to scale). Measurements are averages taken from 1,518 bettong and 432 rabbit pits (Munro et al., 2019).

We created foraging pits that simulated those of bettongs and rabbits, in order to measure changes in pit dimensions (Question 1) and soil chemistry (Question 3) over time. The temperature measurements (Question 2) were taken from real bettong pits (see below). We chose to use artificial pits for two reasons, (1) to be certain of the age of the pits, which is difficult to determine for real pits, and (2) to enable side-by-side comparison of bettong and rabbit pits in the same location and under the same conditions. We created the pits by hand using a teaspoon to scrape and scoop away the soil into a ‘spoil heap’, imitating the action of the animal. We based the size and shape of the pits on measurements of 1,518 bettong and 432 rabbit pits, which were taken previously from the same study site (Munro et al., 2019). While the pits of both animals can vary widely, bettong pits are generally narrower and deeper than rabbit pits with a typical ‘leaning cone’ shape, while rabbit pits are a shallow ‘bowl’ shape (Fig. 1).

### Soil sampling

We took soil samples from six bettong and six rabbit pits in each of the three sites, at 8 months and 24 months after the pits were created (36 pits total at each sampling time). We selected the pits for sampling using a random number generator. In some cases where the pit could not be found or had been disturbed, we used the next suitable pit along the transect. Due to the destructive nature of the sampling, once a pit had been sampled, it could not be sampled again. Sampled pits were also excluded from further measurement for pit dimensions.

For the ‘pit’ sample, we used a small trowel to collect all the loose soil that had accumulated in the pit, down to the original depth indicated by the coloured pebbles. Any large litter was first removed from the site by gently brushing it away. We then sampled the ‘under-pit’ soil below the pebbles using a 50 mL syringe with the end cut off, pushed into the soil at the base of the pit up to the 10 mL mark (Fig. S2). The control samples were taken from an undisturbed area approximately 50 cm further along the transect (i.e. halfway between the pits). For the ‘pit control’, we used the trowel to excavate a new depression of the same dimensions as the paired pit. We then took the ‘under-pit control’ from below the ‘pit control’ sample, using the same method as the ‘under-pit’. This gave a total of 144 samples (36 × pit and under-pit, 36 × pit control and under-pit control).

### Soil analysis

Each soil sample was analysed to measure total nitrogen (N) and total organic carbon (C), mineral nitrogen (NO_3_^−^ and NH_4_^+^), plant-available phosphorus (P), pH and electrical conductivity (EC). The coarsely ground oven-dried soil was finely ground using a puck mill, and the organic carbon and total nitrogen content were determined using a LECO CNS 2000 (C Method 6B2 and N Method 7A5; Rayment & Higginson, 2011). These data were also used to calculate the Carbon:Nitrogen ratio (C:N). A sub-sample of each sample was used to determine soil NO_3_^−^ and NH_4_^+^ content using 1:10 ratio of soil to two M KCl extract. The extract was shaken for 1 h, centrifuged, and filtered prior to analysis. The NO_3_^−^ and NH_4_^+^ concentrations were determined by the cadmium reduction and phenate method (Rice et al., 2012) using an Autoanalyser. Soil plant-available phosphorus was extracted using the Resin P method (Tiessen & Moir, 2007) and determined using the colorimetric molybdate-ascorbic acid method (Murphy & Riley, 1962). Five grams of field soil to 25 ml DI water extract were used to determine soil pH (Method 4A1; Rayment & Higginson, 2011) and EC (Method 3A1; Rayment & Higginson, 2011).

### Temperature measurements

To measure the effect of digging on soil surface temperatures, we selected six real bettong pits at an open grassland site in full sun, within Mulligan’s Flat Nature Reserve. We chose an open site to avoid variation due to shading, so the measurements are likely to represent the most extreme temperature variation experienced in the reserve. These measurements were taken from real bettong pits because we were not concerned about pit age or subsequent disturbance, but we did select pits that appeared to be fresh (i.e. no infill). Pits were randomly distributed across the site, with a minimum distance of one metre between pits. We placed six digital temperature data loggers (Maxim Integrated Thermochron iButton Device DS1921G) in the base of the pits, and six on the soil surface 20 cm from each pit. The thermometers were protected from direct sun by the grass canopy, or a thin layer of loose soil in the bottom of the pits. We set the thermometers to record every 15 min and left them out over 4 days during winter, and again during summer (25–29 Aug and 9–13 Dec 2016).

### Data analysis

To examine the change in physical dimensions of the pits over time (Question 1), we calculated the average radius (length + width/4) and depth of bettong and rabbit artificial pits recorded at 0, 8 and 24 months. We used the ratio of depth to radius as a proxy for the change in the dimensions of the pit. We also calculated the volume of each pit, assuming a circular cone shape (pi * radius^2^ * (depth/3)). We used linear mixed models to test for the interactive effects of pit age and species on the pit dimensions (depth/radius) and volume. We included vegetation type as a random effect to account for site differences. We then tested for pairwise significant differences between the different factor levels using Tukey’s post-hoc test.

To measure the effect of natural bettong pits on soil surface temperatures (Question 2), we plotted the temperatures recorded inside pits and at the soil surface at 15-min intervals, with each interval averaged across the six data loggers for each treatment (‘pit’ and ‘surface’ in summer and winter). We then plotted temperatures as a boxplot to show the overall mean and range for the 4 days of data. For each data logger, we calculated the mean, maximum, minimum and range of temperatures recorded over 4 days in the field. We then conducted paired *t*-tests to test whether there was a difference between the pit and surface in each of summer and winter using GenStat (VSN International, 2015).

To assess the effects of artificial bettong and rabbit pits on soil chemical properties (Question 3), we first used Principle Component Analysis (PCA) to explore the correlations among all the soil variables in relation to other explanatory variables (e.g. vegetation type, age of pit, treatment). This analysis combines variables using an orthogonal transformation to identify compound axes of variation that explain the largest possible variance in the dataset (Pearson, 1901). Eight soil variables were included in the analysis: total nitrogen (N), total carbon (C), C:N ratio, nitrate and ammonium (NO_3_^−^ and NH_4_^+^), plant-available phosphorus (P), pH and EC. We conducted the PCA using PC-ORD (MjM Software Design, 2016).

We next used linear mixed models to test for the interactive effects of our experimental treatments on each of the eight soil variables. Our fixed effects were: *Treatment*—two levels (Treatment and Control), tests for the effect of digging with respect to a paired control (non-pit); *Pit*—two levels (Pit and Under-pit), tests for the difference between soils collected from inside and directly below the pit; *Animal*—two levels (Bettong and Rabbit), tests for the difference between pits created by bettongs or rabbits; *Age*—two levels (8 months and 24 months), tests for the difference due to the age of the pits; *Vegetation type*—three levels (Forest, Woodland and Grassland), tests for the difference due to vegetation type. We were interested in the treatment effect (treatment vs control) in each level of the interaction of age, vegetation type, animal and pit. We used *Pit number* (i.e. position along the transect) as a random effect to account for spatial autocorrelation. Our response variables were Total C (g/kg), Total N (g/kg), C:N ratio, NH_4_^+^ (μg/kg), NO_3_^−^ (μg/kg), P (μg/kg), EC and pH. All response variables were log transformed to achieve normal distribution, except pH. We represented the results of these nested treatment effects as effect sizes. These were extracted from the model coefficients and represent the effect of a treatment vs its corresponding control within each of the interacting effects. We used R (R Core Team, 2017) with the ‘lme4’ (Bates et al., 2015) package for the generalised and linear mixed models, the ‘emmeans’ package (Lenth, 2018) for the Tukey post-hoc test and the ‘ggplot2’ package (Wickham, 2009) for figure plotting (for code see Supplementary Material).

## Results

### Pit dimensions

**Figure 2:**
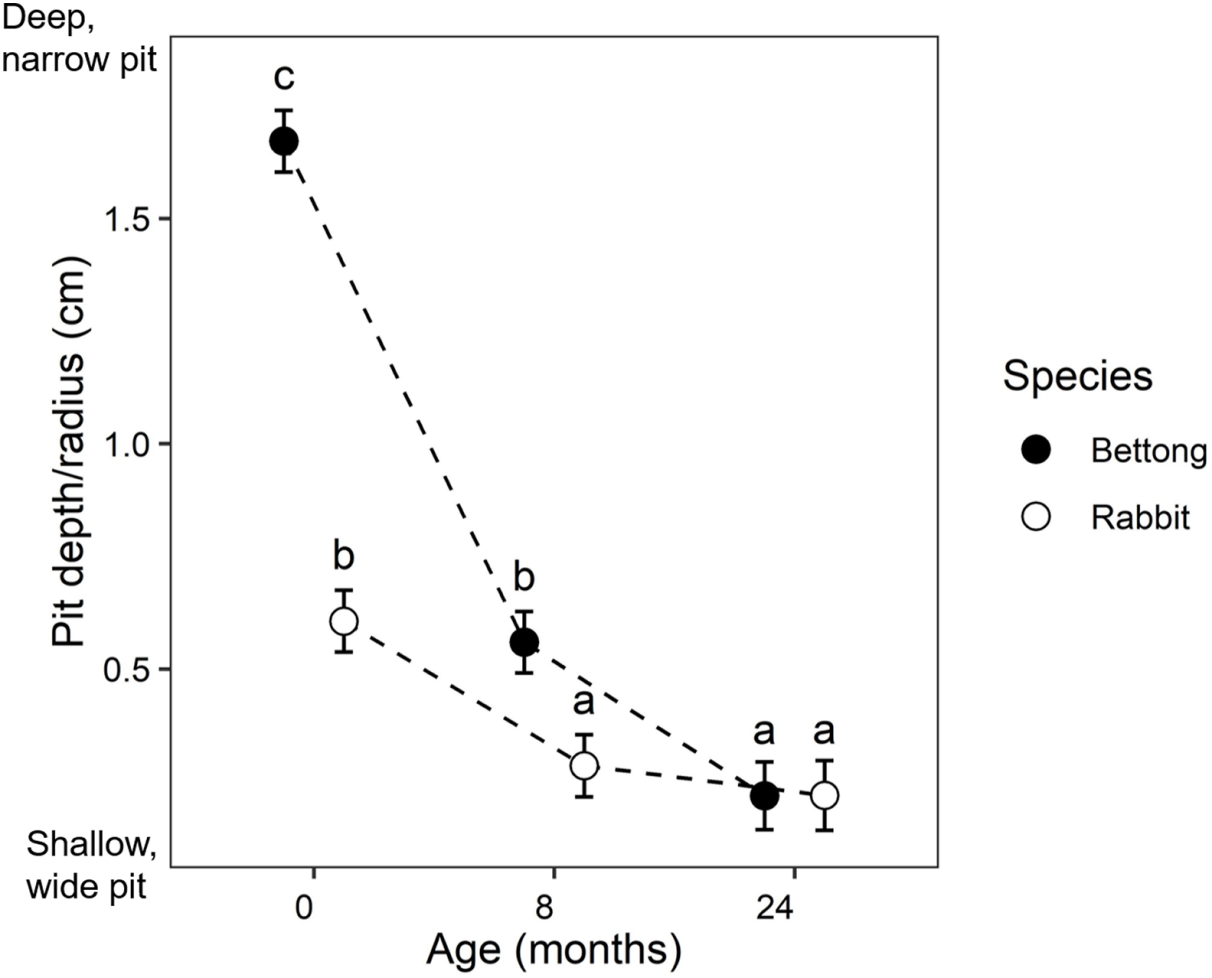
Bettong and rabbit pit dimensions over time, shown as the ratio of pit depth to radius. Values are predicted means with standard errors based on linear mixed models. letters (a–c) indicate pairwise significant differences based on Tukey’s post-hoc test.

We found that after 2 years, 75% of all pits were still visible. After 8 months, 5% of bettong pits were completely filled in, while only 1% of rabbit pits were full. After 24 months, 27% of bettong pits were completely full compared to 17% for rabbit pits. The results of the linear mixed models are shown in Table S1, and the results of the Tukey post-hoc tests are in Table S2. There was a significant interaction between pit age and species (*p* < 0.001) for both depth/radius and volume, indicating that the difference between the bettong and rabbit pits changed over time. At the start of the experiment (0 month), the bettong pits were deeper and narrower than rabbit pits (higher depth/radius ratio), but the rabbit pits had higher average volume due to their larger surface area (see Fig. 2; Fig. S3). At 8 months, the bettong pits had become wider and shallower as their sides collapsed, but the depth/radius ratio was still significantly higher than the rabbit pits. At this point there was no difference in volume between bettong and rabbit pits. Between 8 and 24 months, the bettong pits continued to infill at a slower rate, but there was no significant change in the rabbit pit dimensions. At 24 months there was no difference in dimensions or volume between bettong and rabbit pits.

### Soil surface temperatures

**Figure 3:**
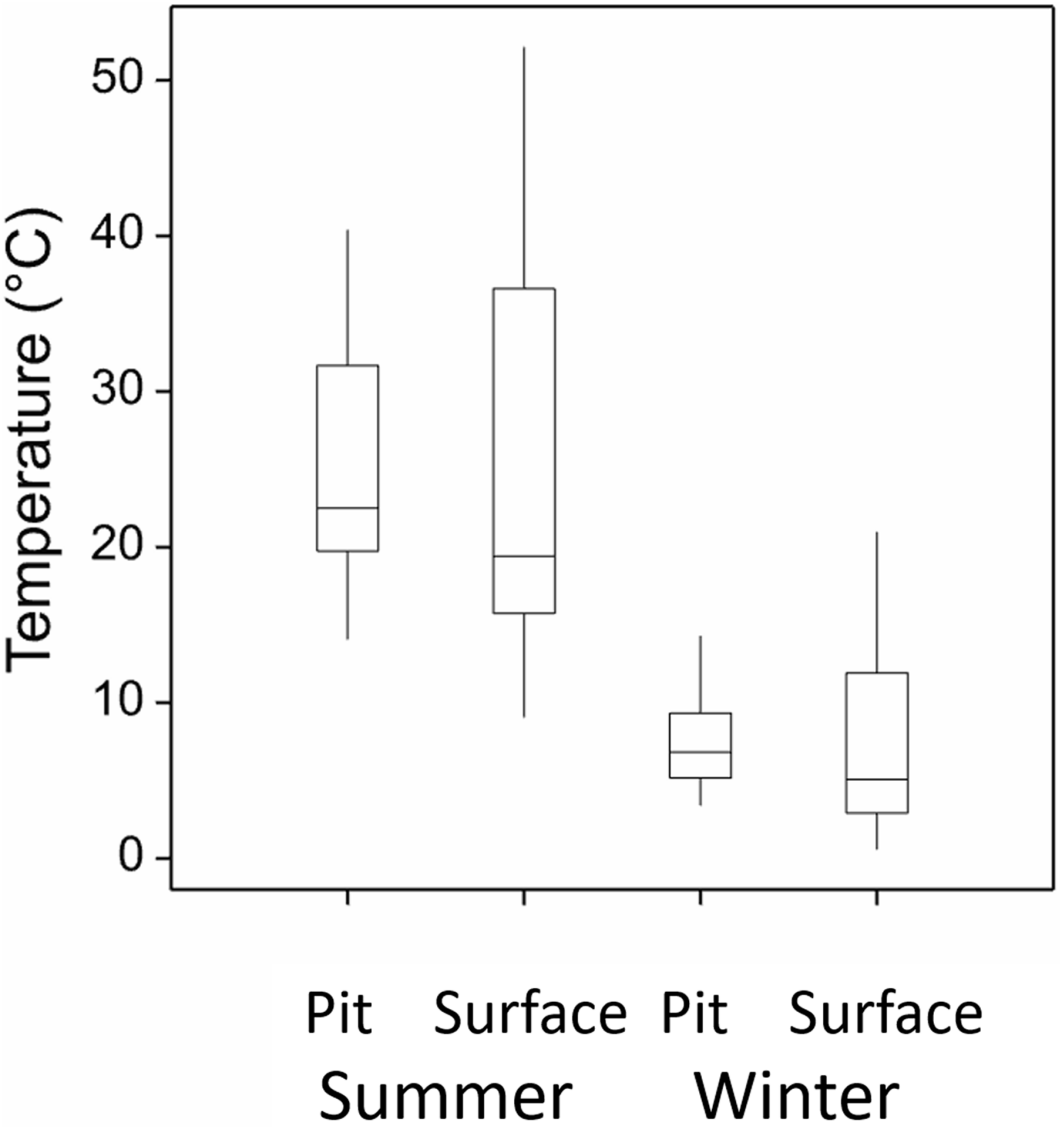
Bettong pit and soil surface temperatures (°C). Temperature data from bettong pits and soil surface, measured every 15 min over 4 days in summer and winter 2016.

There was no difference between the mean temperature in a bettong pit and the soil surface in either summer (*p* = 0.25) or winter (*p* = 0.56) (Fig. 3; Fig. S4). However, the pits were characterised by a significantly smaller diurnal temperature range (summer *p* ≤ 0.001, winter *p* = 0.03). In summer, the mean maximum temperature in a pit was approximately 12 °C cooler than on the surface (*p* ≤ 0.003), the minimum was 3 °C warmer (*p* ≤ 0.001). In winter, the mean maximum temperature in a pit was 5 °C cooler (*p* = 0.03), and the minimum temperature was 2 °C warmer (*p* = 0.02).

### Effect of digging on soil chemistry

**Figure 4:**
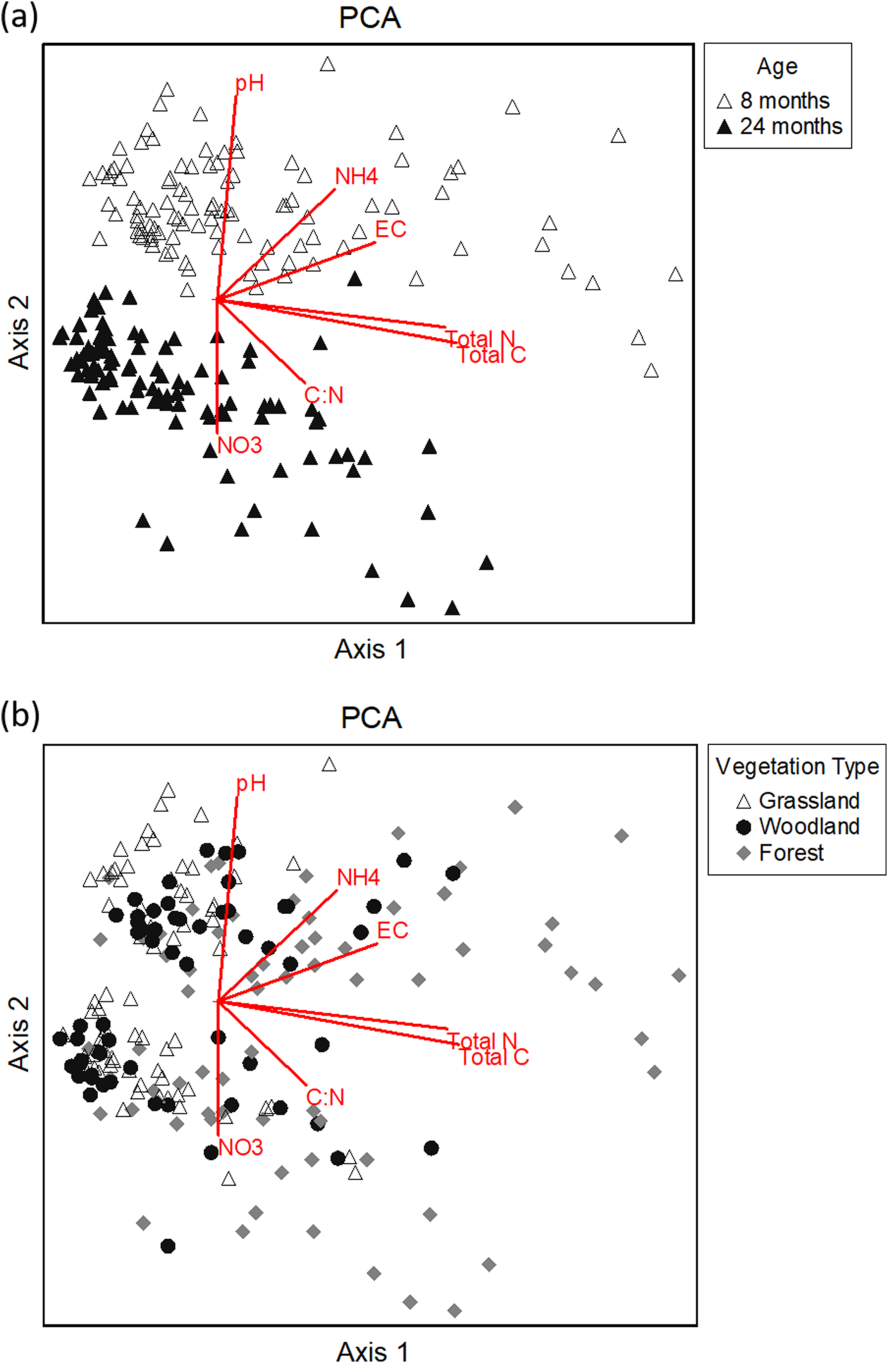
Principle Component Analysis (PCA) of all soil samples. Principal Component Analysis (PCA) of all soil samples (including pit, control, under-pit, and under-pit control), coded by (A) the age of the pit when the samples were taken and (B) vegetation type. The biplot lines indicate direction and strength of correlation with the eight response variables (Total C, Total N, C:N ratio, NH_4_^+^, NO_3_^−^, P, EC and pH).

The PCA showed that our eight soil chemistry variables could be combined into three main axes that explained 77% of the total variation among samples (Table 1). The first axis of the PCA was correlated with C and N (Table 1), and there was a gradient along this axis by vegetation type, with the lowest levels of C and N found in the grassland sites and the highest levels in forest sites (Fig. 4B). Importantly, the PCA ordination revealed that pits were clearly separated by age along the second axis, which was positively correlated with pH and NH_4_^+^, and negatively with NO_3_^−^. At 8 months, soil samples had higher pH and NH_4_^+^, whereas at 24 months the samples tended to have higher levels of NO_3_^−^ (Fig. 4A). There were no obvious visual differences between the bettong and rabbit pits, or between the pit, under-pit and control samples in terms of their positions in ordination space.

**Table 1:** Summary table of the eight soil variables measured for all soil samples (including pit, control, under-pit, and under-pit control), and the results of Principal Components Analysis (PCA) identifying the variation accounted for by the first three axes and their correlated soil variables.

Soil variables	Min.	Max.	Mean	Std. dev.	Correlation with PCA axis		
					Axis 1	Axis 2	Axis 3
Total C g/kg	12.81	413.4	67.41	57.63	0.53	−0.25	−0.16
Total N g/kg	0.6	13.23	3.44	2.25	0.52	−0.20	−0.19
C:N	4.16	37.56	18.46	3.74	0.32	−0.36	−0.15
NO_3_^−^ μg/kg	0.005	90.15	9.16	15.19	0.005	−0.45	0.47
NH_4_^+^ μg/kg	0.02	221.95	29.73	41.63	0.37	0.41	0.006
P μg/kg	0.02	19.49	2.49	3.49	0.09	−0.04	0.81
pH	4.16	6.38	5.17	0.53	0.15	0.56	0.11
EC	4.16	225.6	53.71	40.91	0.43	0.29	0.22
% Variance explained					34.7%	26.7%	15.2%

**Figure 5:**
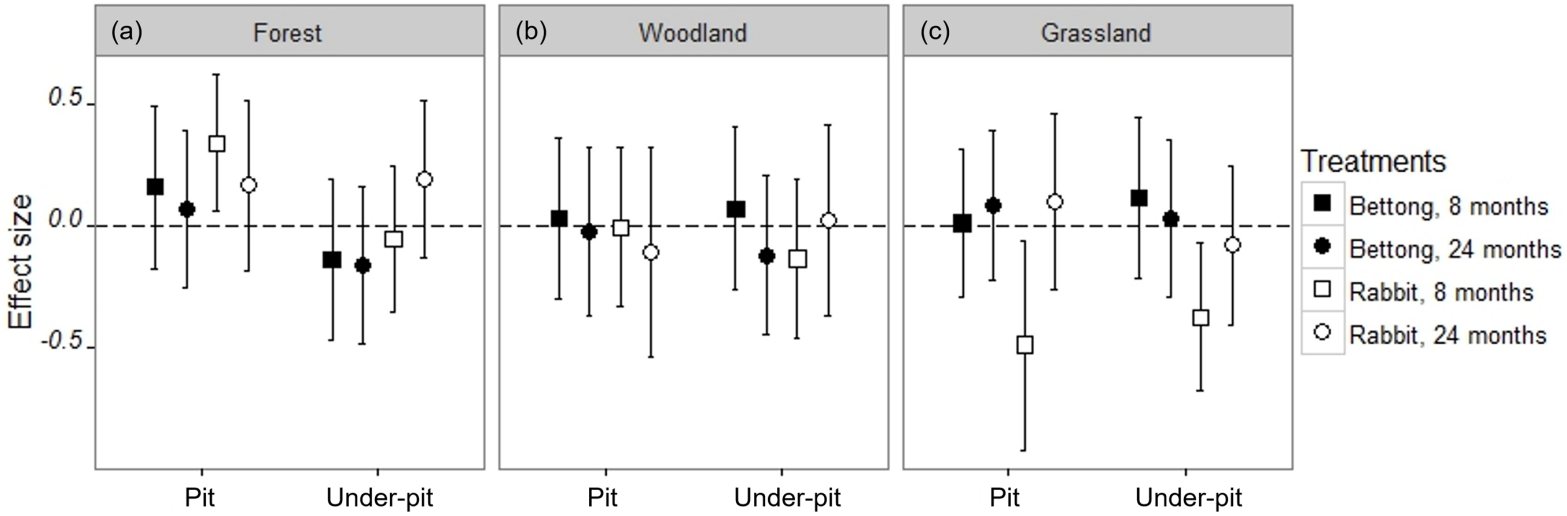
Effect size plot based on linear mixed models for Total Carbon (g/kg). See Figs. S3A–S3H for plots for the other measured soil variables (Total N, C:N ratio, NH_4_^+^, NO_3_^−^, P, EC and pH). These effect sizes are the coefficients of the treatment level vs the control level in the treatment variable. The effects of vegetation type ((A) Forest, (B) Woodland or (C) Grassland), animal (bettong or rabbit), age of pit (8 months or 24 months) and pit vs under-pit on total carbon are all accounted for and not represented in this figure. Points falling above the dotted line indicate a positive effect and below the line is a negative effect. Results are significant only where the confidence intervals do not cross the dotted line.

We found some significant effects of foraging pits on soil chemistry, which are summarised in Table 2 (full results for each variable shown in Figs. S5A–S5H). The effects were dependent on vegetation type, animal, age of pit or some combination thereof, with no consistent patterns across treatments. Most of the significant effects were detected at 8 months, and rabbit pits had more significant effects than bettong pits. For example, rabbit pits in the forest vegetation type had higher levels of carbon at 8 months compared to the control (non-pit), but those in the grassland site had less (Fig. 5); whereas in the woodland, available phosphorus levels were higher in rabbit pits but lower in rabbit under-pits. At 8 months, rabbit pits in the grassland had higher pH, but at 24 months pH was higher in rabbit under-pits in the woodland site. At 24 months, EC in the forest was lower in rabbit pits but higher in the under-pit.

**Table 2:** Summary of results of linear mixed models (LMM), showing effects of digging on eight soil chemistry variables.

	Forest	Woodland	Grassland
Bettong pit	8 months: ↑EC 24 months: NS	8 months: NS 24 months: NS	8 months: ↑pH 24 months: ↑EC
Rabbit pit	8 months: ↑C ↑N 24 months: ↓EC	8 months: ↑P 24 months: NS	8 months: ↓C ↓N ↑pH 24 months: NS
Bettong under-pit	8 months: ↑NH_4_^+^ 24 months: NS	8 months: NS 24 months: NS	8 months: NS 24 months: ↓EC ↓C:N
Rabbit under-pit	8 months: NS 24 months: ↑EC	8 months: ↓P 24 months: NS	8 months: ↓C ↓N 24 months: ↑C:N

**Note:**

Only significant response variables are shown, indicating a significant digging effect when compared with paired controls (non-pit). NS indicates that there were no significant effects.

There were no digging effects on any soil nutrients (C, N, NH_4_^+^, NO_3_^−^ and P) in bettong pits. At 8 months, bettong pits had higher pH in the grassland vegetation type. Bettong pits in the forest had higher EC at 8 months, whereas at 24 months in the grassland EC was higher in bettong pits but lower in the under-pit. In bettong under-pits, ammonium was higher in the forest site at 8 months. There was no significant difference in nitrate among the treatments.

## Discussion

In this study we tested the hypothesis that that foraging pits of an Australian native marsupial, the eastern bettong (*Bettongia gaimardi*), were distinct from control (non-pit) sites and those of the introduced European rabbit in temperate grassy woodlands. We demonstrated that while bettong and rabbit pits differed in their physical effects, such as the micro-topography of the soil surface and the temperatures in the pit, they did not have a clear effect on soil chemistry. We suggest the role of digging ecosystem engineers in temperate zones may be limited to physical disturbance of the soil rather than the creation of nutrient or resource hotspots described previously in arid zone ecosystems. Our work has meaningful implications for understanding the role of ecosystem engineers in temperate ecosystems.

### Question 1—How do the physical dimensions of artificial bettong and rabbit pits change over time?

We found that more than 75% of all pits were still visible after 2 years. This was despite significant rainfall and flooding at around 20 months, which we expected would cause rapid infill. Both pit types filled in more quickly in the first 8 months (Fig. 2) but once they had reached a depth of around one–two cm the rate of infill levelled off, suggesting that they may persist for some time as shallow depressions. Other similar studies have reported decay rates ranging from 4 months up to 3 years depending on vegetation and soil type (Johnson, 1994; Eldridge & Kwok, 2008; Newell, 2008); however, much longer periods have been recorded (see Gutterman, 1987; Whitford & Kay, 1999). We also found that bettong pits filled in slightly faster than rabbit pits. This may be the result of different pit morphology; we observed that the steeper sides of bettong pits quickly collapsed into the hole and appeared to collect more litter and debris, whereas we observed the shallow bowl-shaped rabbit diggings tended to be washed out by wind and water.

### Question 2—Do natural bettong pits influence soil surface temperature?

We found that bettong digging alters physical conditions of the soil surface by moderating the diurnal temperature range up to 25 °C compared to the soil surface. Several previous studies have shown that animal burrows can provide thermal refugia for many species, particularly in arid biomes (Williams, Tieleman & Shobrak, 1999; Casas-Crivillé & Valera, 2005; Read et al., 2008; Walde et al., 2009; Pike & Mitchell, 2013). However, very few studies have measured temperatures in shallow foraging pits. Gutterman (1997) measured temperatures in porcupine pits (~10 cm deep) over a period of two days during spring and summer and found a very similar moderating effect, with daytime temperatures up to 18 °C higher on the soil surface. Eldridge & Mensinga (2007) found that echidna pits (~9 cm deep) were around 2 °C cooler than the soil surface.

### Question 3—What is the effect of artificial bettong and rabbit digging on soil chemistry?

We found that digging influenced soil chemistry, but the effects were not consistent across vegetation types, between bettong and rabbit diggings, or over time. We found the strongest pattern in soil chemistry to be the separation of samples by their different ages (Fig. 4A). This was not an effect of digging but occurred across all samples and was most likely due to seasonal differences in soil moisture and below-ground processes between the sampling times. The 8-month samples were taken in winter, when soil moisture was high, while the 24-month samples were taken in summer and had very low soil moisture. Moisture levels can affect soil pH, EC and particularly the relative concentration of NH_4_^+^ and NO_3_^−^; at high levels of soil moisture, NO_3_^−^ concentration declines while NH_4_^+^ increases (Zhang & Wienhold, 2002). However, the variation in soil moisture would not affect the other variables e.g. total C or N. There also appeared to be a gradient of increasing levels of C and N according to vegetation type (Fig. 4B) and reflects the greater input of organic plant litter in the woodland and forest sites compared to the grassland. The influence of pit age and vegetation type explained most of the variation in chemistry among the samples, making any differences due to digging harder to detect.

We expected that any effects of digging on soil chemistry would change over time, with some changes appearing soon after pit formation, while others may take months or years to develop as the pits fill in. We found that there were more differences in soil chemistry at 8 months after the pits were created, but most of these had disappeared by the second sampling time at 24 months. This suggests that as the pits fill in, they become less distinct from non-pit soil. However, as mentioned above, some of the age effects could have been confounded with seasonal differences. Two years may also be too short a time to observe some effects; most of the pits were not completely filled in, and rates of litter decomposition in this system can be extremely slow due to the inherent low fertility of the soils and the associated leaf traits in the vegetation (Orians & Milewski, 2007; Cornwell et al., 2008; McIntyre et al., 2010).

Our results contrast with the findings of a meta-analysis by Mallen-Cooper, Nakagawa & Eldridge (2019), which found that disturbances greater than 12 months old tended to be more distinct from undisturbed soil than fresh pits. However, ours is the only study we are aware of with repeated sampling over time, and in fact most studies used pits of unknown age. Where the age is known, there is wide variation among studies. For example Travers et al. (2012) found that after 18 months, echidna (*Tachyglossus aculeatus*) pits contained more total C and N than surface soils, whereas Garkaklis, Bradley & Wooller (2003) found a reduction in ammonium, nitrate and sulphur in 3-year-old woylie (*Bettongia penicillata*) diggings but no change in carbon, phosphorus or pH. Parsons et al. (2016) examined pygmy rabbit (*Brachylagus idahoensis*) burrows and found that duration of occupancy (1–12 years) had a limited effect on soil nutrients.

### General discussion

Our results support, in part, the hypothesis that the importance of ecosystem engineers differs across gradients of environmental stress (Crain & Bertness, 2006). Most studies of digging animals in Australia have been in arid or semi-arid biomes (Coggan, Hayward & Gibb, 2018), and a recent global review of digging animals found that soil disturbance effects were generally stronger in more arid environments (Mallen-Cooper, Nakagawa & Eldridge, 2019). Several studies have suggested that this is due to the creation of resource ‘hotspots’, where pits collect litter and moisture and become concentrated patches of these limited resources (Eldridge & Mensinga, 2007; Eldridge & Whitford, 2009; James, Eldridge & Hill, 2009). We suggest that in our temperate woodland system, resources like water and nutrients are not as limiting and may be more evenly distributed across the landscape, so any difference between dug and un-dug patches is likely to be less pronounced. In more benign or mesic environments, competition becomes more of a limiting factor (Crain & Bertness, 2006), so the removal of existing vegetation and creation of gaps may be more important for some species (e.g. gap-dependent forbs (Grubb, 1977; Morgan, 1998)) than the provision of resources.

The eastern bettong pits measured at Mulligan’s Flat are also considerably smaller and shallower than the pits of other species such as the bilby or the burrowing bettong (Newell, 2008) or those of the same species recorded in Tasmanian dry sclerophyll forest (Davies et al., 2019), so they may not be as effective at incorporating organic matter into the deeper layers of soil. The reason for this difference in pit size is unclear, but could be due to differences in soil type, depth, moisture or compaction making it harder to dig, the availability of food at different depths, or the fact that bettongs and other digging animals have long been absent from the site. This would be an interesting avenue for further research.

According to the framework put forward in Jones et al. (2010), the magnitude of structural change created by an ecosystem engineer is a function of the rate of structure formation and the rate of decay i.e. how long the structure persists without maintenance. A previous study at the same site estimated the rate of digging by bettongs, rabbits and other digging animals (Munro et al., 2019). However, it was limited to a short timeframe and did not measure decay rates. Our study therefore adds to our understanding of the persistence of the effects of digging animals in ecosystems.

Pit longevity, morphology and microclimate may have other ecological implications. Studies have shown that animal diggings provide sites for seed germination, particularly in arid environments (Gutterman & Herr, 1981; James, Eldridge & Moseby, 2010; Valentine et al., 2017). While we did not investigate impacts of digs on other biota in this study, we did observe seedlings germinating in pits. James et al. (2011) found that pits of native marsupials contained 80% more seedlings than rabbit pits, which they attributed to the difference in morphology. The steeper sided bettong pits may also make it more difficult for ants to remove seeds (Radnan & Eldridge, 2017). While bettong pits are too small to provide habitat for most vertebrates, the temperature moderating effect may be important for generating heterogeneity in microclimate for seedlings, microbes and some invertebrates (Eldridge & Mensinga, 2007; James, Eldridge & Hill, 2009). We observed that pits were often free from frost in winter and appeared to retain moisture longer after rainfall events in summer. Further research is needed to confirm whether pits have an impact on seed germination and other biota in temperate grassy woodland and the mechanisms driving this effect.

It is important to note that because we used artificial pits, they may not fully replicate the effects of a real bettong or rabbit digging. Artificial pits do not capture the wide range of natural variation in size and shape, which may depend on soil type, time of year and many other factors. By using artificial pits, we expected to reduce this variation to detect differences between treatments more easily. Natural pits may also have unknown qualities, for example it has also been suggested that bettongs and other mycophagous species may be able to spread fungal spores via their noses or in their faeces (Claridge et al., 1992; Martin, 2003), and this of course cannot be replicated with artificial pits.

While rabbits are considered pests in Australia, it has been suggested that they could fill the niche created by the loss of native engineers (Read et al., 2008; James et al., 2011). We found that the morphology of bettong and rabbit diggings had an impact on their infill rate and longevity, with rabbit diggings taking longer to fill than bettong diggings. This difference in dig morphology may mean that rabbit diggings are not able to fully replicate the ecosystem engineering effects of the native bettong. Previous research by Munro et al. (2019) found that bettongs have a much higher rate of soil turnover than either rabbits or other common native species such as echidnas or ground-foraging birds (e.g. white-winged chough, *Corcorax melanorhamphos*). Rabbits have also famously shown the explosive population dynamics that sometimes occur with species introduced into a new range, with devastating impacts on native species and ecosystems (Eldridge & Simpson, 2002; Johnson, 2006). However, in areas where other native diggers have disappeared, rabbits (or other exotic species such as pigs) may be the only digging species remaining, and this should be considered before undertaking rabbit control programmes where there are no native digging species present. Ideally, replacement of introduced diggers by native diggers in an integrated restoration programme would be the preferred solution to this.

## Conclusions

We examined the effects of foraging pits of the eastern bettong and introduced rabbits on soil physical and chemical properties in a temperate grassy woodland ecosystem. We found that pits of bettong and rabbit pits differed in their morphology and longevity and that bettong pits moderated daily temperature extremes. We also found that more than 75% of all pits were still visible after 2 years. However, digging did not have consistent effects on soil chemistry. These results differ from those found in arid ecosystems and suggest the effects of ecosystem engineers in temperate grassy woodlands are restricted to physical alteration of the soil rather than the creation of nutrient hotspots.

## Supplemental Information

10.7717/peerj.7506/supp-1 Supplemental Information 1 Figures S1–S5, Tables S1–S2 and R code used for analyses and plots. Figure S1. Monthly rainfall totals for Canberra over the experimental period (Nov 2014–Feb 2017). Figure S2. Method used to collect ‘under-pit’ samples. Figure S3. Volume of bettong and rabbit pits over time. Figure S4. Bettong pit and soil surface temperature (°C) in summer and winter. Figure S5 (A–H). Effect size plots based on linear mixed models for all eight soil variables (Total C, Total N, C:N ratio, NH4^+^, NO_3_^−^, P, EC and pH). Table S1. Summary of results of linear mixed models showing change in pit dimensions and volume over time. Table S2. Summary of results of Tukey’s post-hoc tests, based on predicted responses from the linear mixed models for pit dimensions and volume (see Table S1).

10.7717/peerj.7506/supp-2 Supplemental Information 2 Pit dimensions.

10.7717/peerj.7506/supp-3 Supplemental Information 3 Temperature raw data.

10.7717/peerj.7506/supp-4 Supplemental Information 4 Soil raw data.

## Additional Information and Declarations

### Data Availability

The following information was supplied regarding data availability: The raw data and code are available in the Supplemental Files.

## References

[ref-1] Alkon PU (1999). Microhabitat to landscape impacts: crested porcupine digs in the Negev Desert highlands. Journal of Arid Environments.

[ref-85] Australian Government (2006). EPBC Act.

[ref-2] Bates D, Maechler M, Bolker BM, Walker S (2015). lme4: Linear mixed-effects models using Eigen and S4. Journal of Statistical Software.

[ref-3] Batson WG, Gordon IJ, Fletcher DB, Manning AD (2016). The effect of pre-release captivity on post-release performance in reintroduced eastern bettongs Bettongia gaimardi. Oryx.

[ref-4] Berke SK (2010). Functional groups of ecosystem engineers: a proposed classification with comments on current issues. Integrative and Comparative Biology.

[ref-5] Burbidge AA, McKenzie NL (1989). Patterns in the modern decline of western Australia’s vertebrate fauna: causes and conservation implications. Biological Conservation.

[ref-86] Bureau of Meteorology (2018). Weather and climate data.

[ref-6] Byers JE, Cuddington K, Jones CG, Talley TS, Hastings A, Lambrinos JG, Crooks JA, Wilson WG (2006). Using ecosystem engineers to restore ecological systems. Trends in Ecology & Evolution.

[ref-7] Canals RM, Herman DJ, Firestone MK (2003). How disturbance by fossorial mammals alters N cycling in a California annual grassland. Ecology.

[ref-8] Casas-Crivillé A, Valera F (2005). The European bee-eater (Merops apiaster) as an ecosystem engineer in arid environments. Journal of Arid Environments.

[ref-9] Claridge AW, Tanton MT, Seebeck JH, Cork SJ, Cunningham RB (1992). Establishment of ectomycorrhizae on the roots of two species of *Eucalyptus* from fungal spores contained in the faeces of the long-nosed potoroo (Potorous tridactylus). Austral Ecology.

[ref-10] Coggan NV, Hayward MW, Gibb H (2016). Termite activity and decomposition are influenced by digging mammal reintroductions along an aridity gradient. Journal of Arid Environments.

[ref-11] Coggan NV, Hayward MW, Gibb H (2018). A global database and ‘state of the field’ review of research into ecosystem engineering by land animals. Journal of Animal Ecology.

[ref-12] Cornwell WK, Cornelissen JHC, Amatangelo K, Dorrepaal E, Eviner VT, Godoy O, Hobbie SE, Hoorens B, Kurokawa H, Pérez-Harguindeguy N, Quested HM, Santiago LS, Wardle DA, Wright IJ, Aerts R, Allison SD, van Bodegom P, Brovkin V, Chatain A, Callaghan TV, Díaz S, Garnier E, Gurvich DE, Kazakou E, Klein JA, Read J, Reich PB, Soudzilovskaia NA, Vaieretti MV, Westoby M (2008). Plant species traits are the predominant control on litter decomposition rates within biomes worldwide. Ecology Letters.

[ref-13] Crain CM, Bertness MD (2006). Ecosystem engineering across environmental gradients: implications for conservation and management. BioScience.

[ref-14] Cuevas MF, Mastrantonio L, Ojeda RA, Jaksic FM (2012). Effects of wild boar disturbance on vegetation and soil properties in the Monte Desert, Argentina. Mammalian Biology.

[ref-15] Davies GTO, Kirkpatrick JB, Cameron EZ, Carver S, Johnson CN (2019). Ecosystem engineering by digging mammals: effects on soil fertility and condition in Tasmanian temperate woodland. Royal Society Open Science.

[ref-16] Decker O, Eldridge DJ, Gibb H (2019). Restoration potential of threatened ecosystem engineers increases with aridity: broad scale effects on soil nutrients and function. Ecography.

[ref-17] Eldridge DJ (2004). Mounds of the American Badger (*Taxidea Taxus*): significant features of North American shrub-steppe ecosystems. Journal of Mammalogy.

[ref-18] Eldridge DJ (2009). Badger (Taxidea taxus) mounds affect soil hydrological properties in a degraded shrub-steppe. American Midland Naturalist.

[ref-19] Eldridge DJ, Delgado-Baquerizo M, Woodhouse JN, Neilan BA (2016). Mammalian engineers drive soil microbial communities and ecosystem functions across a disturbance gradient. Journal of Animal Ecology.

[ref-20] Eldridge DJ, Huang N, Bentley J, Hayward MW (2012a). Soil disturbance by invertebrates in a semi-arid eucalypt woodland: effects of grazing exclusion, faunal reintroductions, landscape and patch characteristics. Proceedings of the Linnean Society of New South Wales.

[ref-21] Eldridge DJ, James AI (2009). Soil-disturbance by native animals plays a critical role in maintaining healthy Australian landscapes. Ecological Management & Restoration.

[ref-22] Eldridge DJ, Koen TB (2008). Formation of nutrient-poor soil patches in a semi-arid woodland by the European rabbit (*Oryctolagus cuniculus L*.). Austral Ecology.

[ref-23] Eldridge DJ, Koen TB, Killgore A, Huang N, Whitford WG (2012b). Animal foraging as a mechanism for sediment movement and soil nutrient development: Evidence from the semi-arid Australian woodlands and the Chihuahuan Desert. Geomorphology.

[ref-24] Eldridge DJ, Kwok ABC (2008). Soil disturbance by animals at varying spatial scales in a semi-arid Australian woodland. Rangeland Journal.

[ref-25] Eldridge DJ, Mensinga A (2007). Foraging pits of the short-beaked echidna (Tachyglossus aculeatus) as small-scale patches in a semi-arid Australian box woodland. Soil Biology and Biochemistry.

[ref-26] Eldridge DJ, Myers CA (2001). The impact of warrens of the European rabbit (*Oryctolagus cuniculus* L.) on soil and ecological processes in a semi-arid Australian woodland. Journal of Arid Environments.

[ref-27] Eldridge DJ, Simpson R (2002). Rabbit (*Oryctolagus cuniculus* L.) impacts on vegetation and soils, and implications for management of wooded rangelands. Basic and Applied Ecology.

[ref-28] Eldridge DJ, Whitford WG (2009). Badger (*Taxidea taxus*) disturbances increase soil heterogeneity in a degraded shrub-steppe ecosystem. Journal of Arid Environments.

[ref-29] Eldridge DJ, Woodhouse JN, Curlevski NJA, Hayward M, Brown MV, Neilan BA (2015). Soil-foraging animals alter the composition and co-occurrence of microbial communities in a desert shrubland. ISME Journal.

[ref-30] Fleming PA, Anderson H, Prendergast AS, Bretz MR, Valentine LE, Hardy GESJ (2014). Is the loss of Australian digging mammals contributing to a deterioration in ecosystem function?. Mammal Review.

[ref-31] Gálvez-Bravo L, López-Pintor A, Rebollo S, Gómez-Sal A (2011). European rabbit (*Oryctolagus cuniculus*) engineering effects promote plant heterogeneity in Mediterranean dehesa pastures. Journal of Arid Environments.

[ref-32] Garkaklis MJ, Bradley JS, Wooller RD (1998). The effects of Woylie (*Bettongia penicillata*) foraging on soil water repellency and water infiltration in heavy textured soils in southwestern Australia. Austral Ecology.

[ref-33] Garkaklis MJ, Bradley JS, Wooller RD (2003). The relationship between animal foraging and nutrient patchiness in south-west Australian woodland soils. Australian Journal of Soil Research.

[ref-34] Groot Bruinderink GWTA, Hazebroek E (1996). Wild boar (*Sus scrofa scrofa* L.) rooting and forest regeneration on podzolic soils in the Netherlands. Forest Ecology and Management.

[ref-35] Grubb PJ (1977). The maintenance of species richness in plant communities: the importance of the regeneration niche. Biological Reviews.

[ref-36] Gutterman Y (1987). Dynamics of porcupine (Hystrix indica Kerr) diggings: their role in the survival and renewal of geophytes and hemicryptophytes in the Negev Desert highlands. Israel Journal of Botany.

[ref-37] Gutterman Y (1997). Spring and summer daily subsurface temperatures in three microhabitats in a flat natural loess area in the Negev Desert, Israel. Journal of Arid Environments.

[ref-38] Gutterman Y, Herr N (1981). Influences of porcupine (*Hystrix indica*) activity on the slopes of the northern Negev mountains: germination and vegetation renewal in different geomorphological types and slope directions. Oecologia.

[ref-39] Hastings A, Byers JE, Crooks JA, Cuddington K, Jones CG, Lambrinos JG, Talley TS, Wilson WG (2007). Ecosystem engineering in space and time. Ecology Letters.

[ref-40] Hoekstra JM, Boucher TM, Ricketts TH, Roberts C (2004). Confronting a biome crisis: global disparities of habitat loss and protection. Ecology Letters.

[ref-41] James AI, Eldridge DJ (2007). Reintroduction of fossorial native mammals and potential impacts on ecosystem processes in an Australian desert landscape. Biological Conservation.

[ref-42] James AI, Eldridge DJ, Hill BM (2009). Foraging animals create fertile patches in an Australian desert shrubland. Ecography.

[ref-43] James AI, Eldridge DJ, Koen TB, Moseby KE (2011). Can the invasive European rabbit (*Oryctolagus cuniculus*) assume the soil engineering role of locally-extinct natives?. Biological Invasions.

[ref-44] James AI, Eldridge DJ, Moseby KE (2010). Foraging pits, litter and plant germination in an arid shrubland. Journal of Arid Environments.

[ref-45] Johnson CN (1994). Distribution of feeding activity of the Tasmanian bettong (Bettongia gaimardi) in relation to vegetation patterns. Wildlife Research.

[ref-46] Johnson CN (2006). Interactions: rabbits, sheep and dingoes. Australia’s Mammal Extinctions—a 50 000 Year History.

[ref-47] Jones CG, Gutiérrez JL, Byers JE, Crooks JA, Lambrinos JG, Talley TS (2010). A framework for understanding physical ecosystem engineering by organisms. Oikos.

[ref-48] Jones CG, Lawton JH, Shachak M (1994). Organisms as ecosystem engineers. Oikos.

[ref-49] Kinlaw A (1999). A review of burrowing by semi-fossorial vertebrates in arid environments. Journal of Arid Environments.

[ref-50] Laundre JW (1993). Effects of small mammal burrows on water infiltration in a cool desert environment. Oecologia.

[ref-51] Lenth R (2018). emmeans: estimated marginal means, aka least-squares means. https://cran.r-project.org/web/packages/emmeans/index.html.

[ref-52] Lepschi BJ (1993). Vegetation of Mulligans Flat, A.C.T. Cunninghamia.

[ref-53] Mallen-Cooper M, Nakagawa S, Eldridge DJ (2019). Global meta-analysis of soil-disturbing vertebrates reveals strong effects on ecosystem patterns and processes. Global Ecology and Biogeography.

[ref-54] Manning AD, Eldridge DJ, Jones CG, Armstrong D, Hayward M, Moro D, Seddon P (2015). Policy implications of ecosystem engineering for multiple ecosystem benefits. Advances in Reintroduction Biology of Australian and New Zealand Fauna.

[ref-55] Manning A, Wood J, Cunningham R, McIntyre S, Shorthouse D, Gordon I, Lindenmayer D (2011). Integrating research and restoration: the establishment of a long-term woodland experiment in south-eastern Australia. Australian Zoologist.

[ref-56] Martin G (2003). The role of small ground-foraging mammals in topsoil health and biodiversity: implications to management and restoration. Ecological Management and Restoration.

[ref-57] McIntyre S, Stol J, Harvey J, Nicholls AO, Campbell M, Reid A, Manning AD, Lindenmayer DB (2010). Biomass and floristic patterns in the ground layer vegetation of box-gum grassy eucalypt woodland in Goorooyarroo and Mulligans Flat Nature Reserves, Australian Capital Territory. Cunninghamia.

[ref-58] MjM Software Design (2016). https://www.wildblueberrymedia.net/pcord.

[ref-59] Morgan JW (1998). Importance of canopy gaps for recruitment of some forbs in Themeda triandra-dominated grasslands in South-eastern Australia. Australian Journal of Botany.

[ref-60] Munro NT, McIntyre S, Macdonald B, Cunningham SA, Gordon IJ, Cunningham RB, Manning AD (2019). Returning a lost process by reintroducing a locally extinct digging marsupial. PeerJ.

[ref-61] Murphy J, Riley JP (1962). A modified single solution method for the determination of phosphate in natural waters. Analytica Chimica Acta.

[ref-62] Newell J (2008). The role of the reintroduction of Greater Bilbies (Macrotis lagotis) and Burrowing Bettongs (Bettongia lesueur) in the ecological restoration of an arid ecosystem: foraging diggings, diet and soil seed banks.

[ref-63] Orians GH, Milewski AV (2007). Ecology of Australia: the effects of nutrient-poor soils and intense fires. Biological Reviews.

[ref-64] Parsons MA, Barkley TC, Rachlow JL, Johnson-Maynard JL, Johnson TR, Milling CR, Hammel JE, Leslie I, Perring M (2016). Cumulative effects of an herbivorous ecosystem engineer in a heterogeneous landscape. Ecosphere.

[ref-65] Pearson K (1901). LIII. On lines and planes of closest fit to systems of points in space. The London, Edinburgh, and Dublin Philosophical Magazine and Journal of Science.

[ref-66] Pike DA, Mitchell JC (2013). Burrow-dwelling ecosystem engineers provide thermal refugia throughout the landscape. Animal Conservation.

[ref-67] Radnan GN, Eldridge DJ (2017). Does the morphology of animal foraging pits influence secondary seed dispersal by ants?. Austral Ecology.

[ref-68] Rayment GE, Higginson FR (2011). Soil chemical methods: Australasia.

[ref-69] Raynaud X, Jones CG, Barot S (2013). Ecosystem engineering, environmental decay and environmental states of landscapes. Oikos.

[ref-70] R Core Team (2017). R: a language and environment for statistical computing.

[ref-71] Read JL, Carter J, Moseby KE, Greenville A (2008). Ecological roles of rabbit, bettong and bilby warrens in arid Australia. Journal of Arid Environments.

[ref-87] Rice EW, Baird RB, Eaton AD, Clesceri LS (2012). Standard Methods for the Examination of Water and Wastewater.

[ref-72] Short J (1998). The extinction of rat-kangaroos (Marsupialia: Potoroidae) in New South Wales. Biological Conservation.

[ref-73] Short J, Smith A (1994). Mammal decline and recovery in Australia. Journal of Mammalogy.

[ref-74] Shorthouse DJ, Iglesias D, Jeffress S, Lane S, Mills P, Woodbridge G, McIntyre S, Manning AD (2012). The ‘making of’ the Mulligans Flat—Goorooyarroo experimental restoration project. Ecological Management & Restoration.

[ref-75] Tardiff SE, Stanford JA (1998). Grizzly bear digging: effects on subalpine meadow plants in relation to mineral nitrogen availability. Ecology.

[ref-76] Tiessen H, Moir JO, Carter MR, Gregorich EG (2007). Characterization of available P by sequential extraction. Soil Sampling and Methods of Analysis.

[ref-77] Travers SK, Eldridge DJ, Koen TB, Soliveres S (2012). Animal foraging pit soil enhances the performance of a native grass under stressful conditions. Plant and Soil.

[ref-78] Valentine LE, Bretz M, Ruthrof KX, Fisher R, Hardy GESJ, Fleming PA (2017). Scratching beneath the surface: Bandicoot bioturbation contributes to ecosystem processes. Austral Ecology.

[ref-79] VSN International (2015). GenStat for Windows.

[ref-80] Walde AD, Walde AM, Delaney DK, Fater LL (2009). Burrows of desert tortoises (Gopherus agassizii) as thermal refugia for horned larks (Eremophila alpestris) in the Mojave Desert. Southwestern Naturalist.

[ref-81] Whitford WG, Kay FR (1999). Biopedturbation by mammals in deserts: a review. Journal of Arid Environments.

[ref-82] Wickham H (2009). ggplot2: elegant graphics for data analysis.

[ref-83] Williams JB, Tieleman BI, Shobrak M (1999). Lizard burrows provide thermal refugia for larks in the Arabian desert. Condor.

[ref-84] Zhang R, Wienhold BJ (2002). The effect of soil moisture on mineral nitrogen, soil electrical conductivity, and pH. Nutrient Cycling in Agroecosystems.

