# Supplementary material for: Effects of digging by a native and introduced ecosystem engineer on soil physical and chemical properties in temperate grassy woodland
Source: PeerJ. 2019 Aug 20;7:e7506. doi: 10.7717/peerj.7506 (PMC6710926; doi:10.7717/peerj.7506)
Supplement: Supplemental Information 1 — Figure S1. Monthly rainfall totals for Canberra over the experimental period (Nov 2014–Feb 2017). Figure S2. Method used to collect ‘under-pit’ samples. Figure S3. Volume of bettong and rabbit pits over time. Figure S4. Bettong pit and soil surface temperature (°C) in summer and winter. Figure S5 (A–H). Effect size plots based on linear mixed models for all eight soil variables (Total C, Total N, C:N ratio, NH4+, NO3−, P, EC and pH). Table S1. Summary of results of linear mixed models showing change in pit dimensions and volume over time. Table S2. Summary of results of Tukey’s post-hoc tests, based on predicted responses from the linear mixed models for pit dimensions and volume (see Table S1). [file peerj-07-7506-s001.docx]

# Supplementary material

# Effects of digging by a native and introduced ecosystem engineer on soil physical and chemical properties in temperate grassy woodland

Catherine E. Ross, Nicola T. Munro, Philip S. Barton, M. John Evans, John Gillen, Bennett C. T. Macdonald, Sue McIntyre, Saul A. Cunningham, Adrian D. Manning

**Figure S1**. Monthly rainfall (in mm) totals for Canberra over the experimental period (Nov 2014- Feb 2017). Artificial pits were established in December 2014, and sampling was conducted in August 2015 and January 2017 (indicated with arrows). Data sourced from the Australian Government Bureau of Meteorology (<http://www.bom.gov.au/>) ‘Ginninderra CSIRO’ weather station.


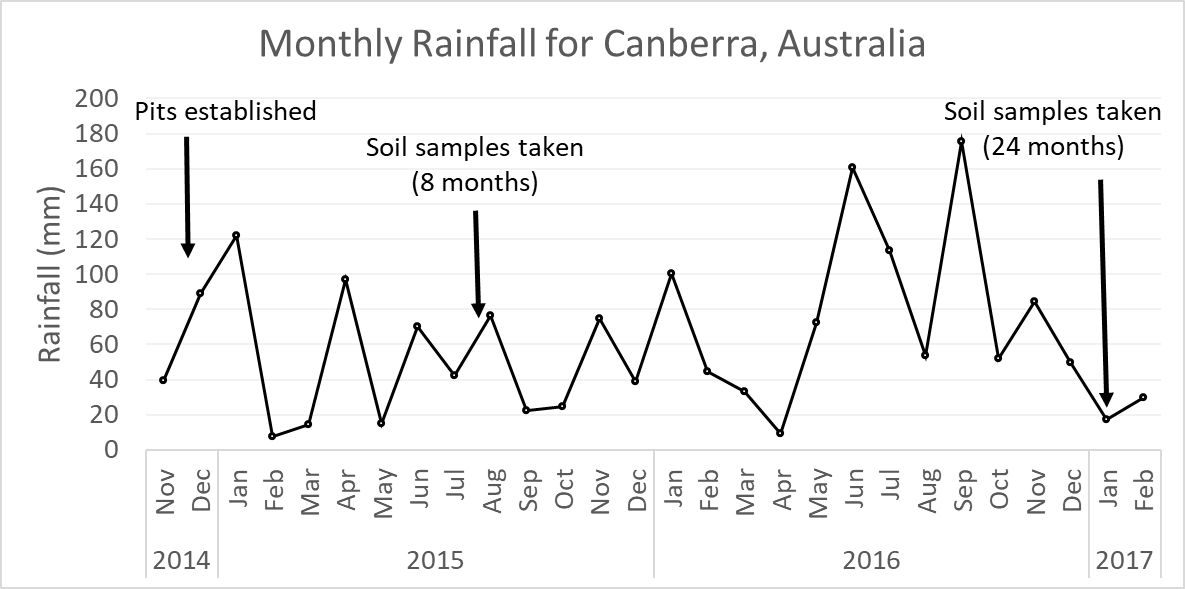


**Figure S2.** Method used to collect ‘under-pit’ samples – a 50mL syringe with the end cut off is pushed into the soil at the base of the pit up to the 10mL mark, giving a sample of approximately 10mL.


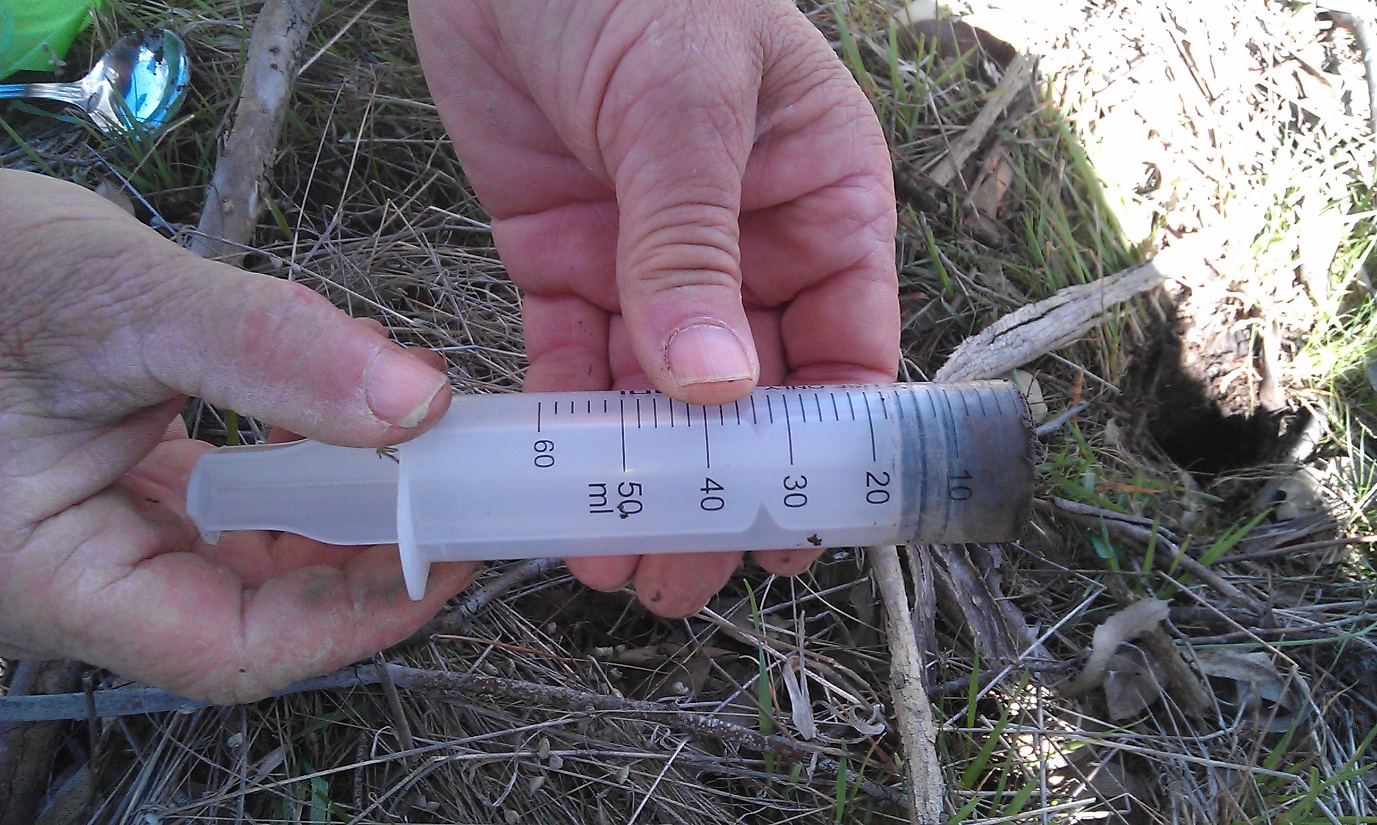


**Figure S3.** Volume of bettong and rabbit pits over time. Values are predicted means with standard errors based on linear mixed models. Letters (a-e) indicate pairwise significant differences based on Tukey’s post-hoc test.

**
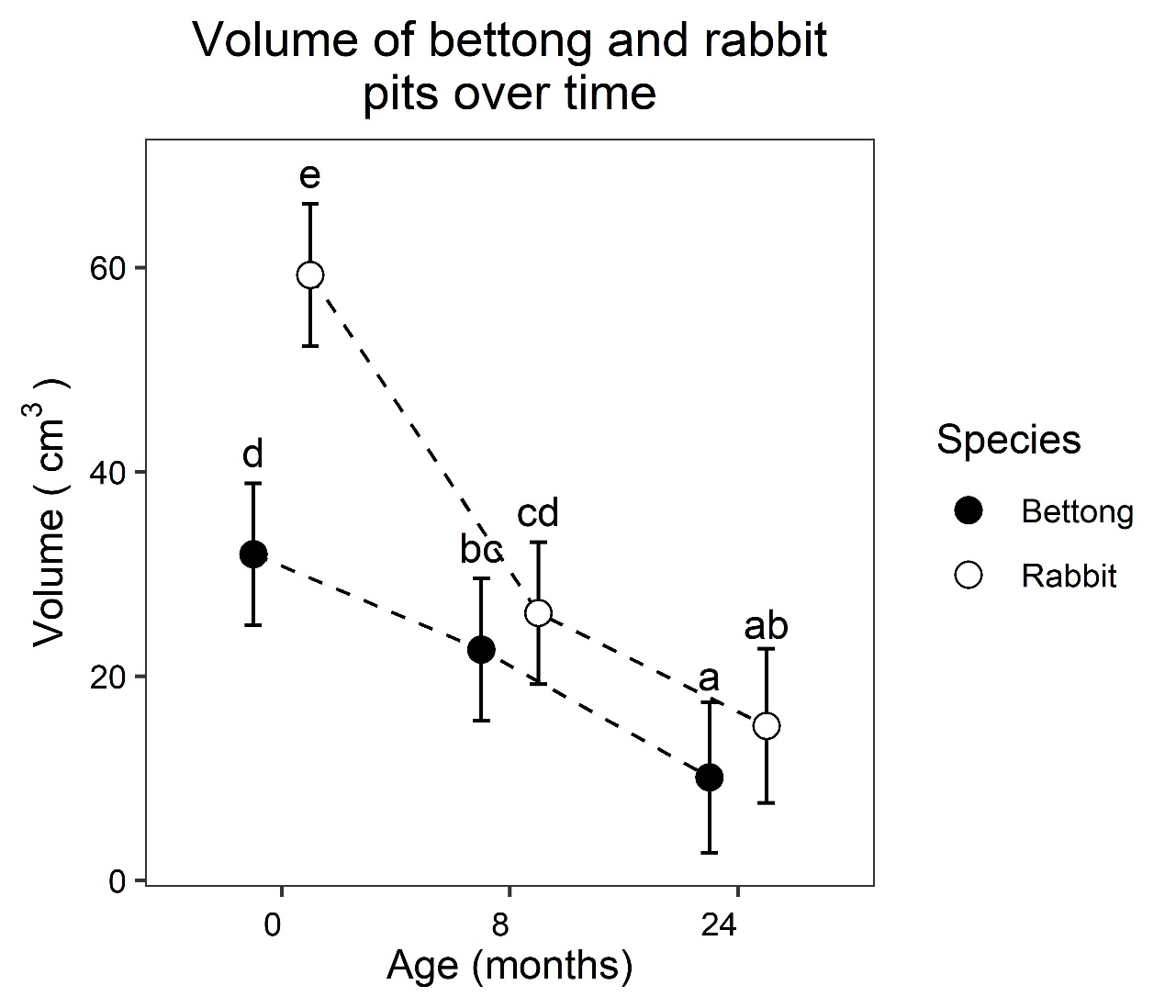
**

**Figure S4.** Bettong pit and soil surface temperature (°C) in summer and winter. Temperature at soil surface and at bottom of bettong pits, averaged across six thermometers for each treatment. Measured every 15 minutes over four days in summer and winter 2016.


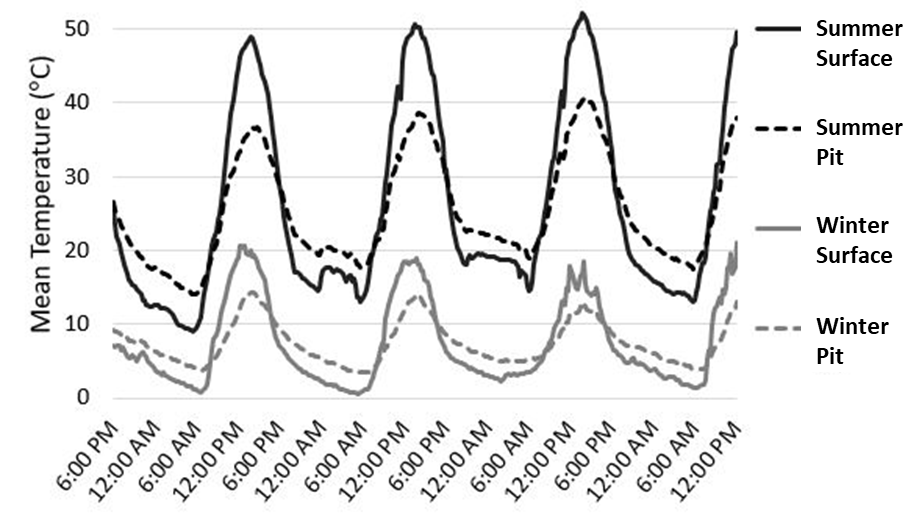


**Figure S5 (a-h)**. Effect size plots based on linear mixed models for all eight soil variables (Total C, Total N, C:N ratio, NH_4_+, NO_3_-, P, EC and pH). Plots show the treatment effects (pit vs non-pit) after taking into account vegetation type, animal, age of pit, and pit vs under-pit, and their interactions. Points falling above the dotted line indicate a positive effect and below the line is a negative effect. Results are significant only where the confidence intervals do not cross the dotted line.

1. Total C g/kg


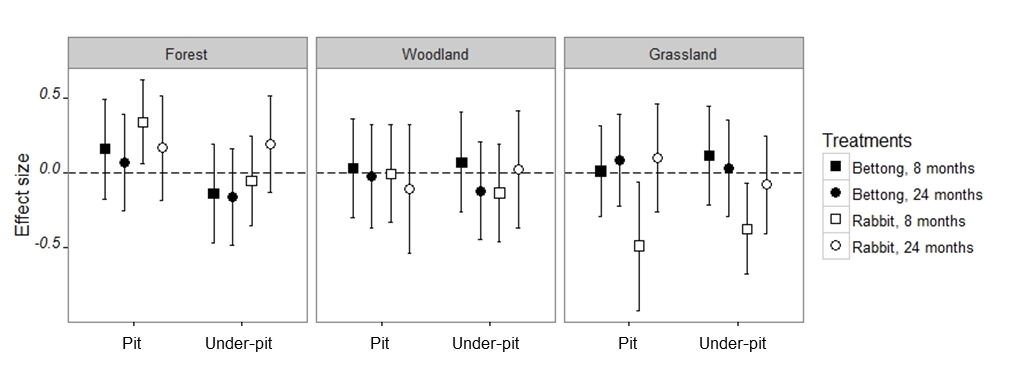


1. Total N g/kg


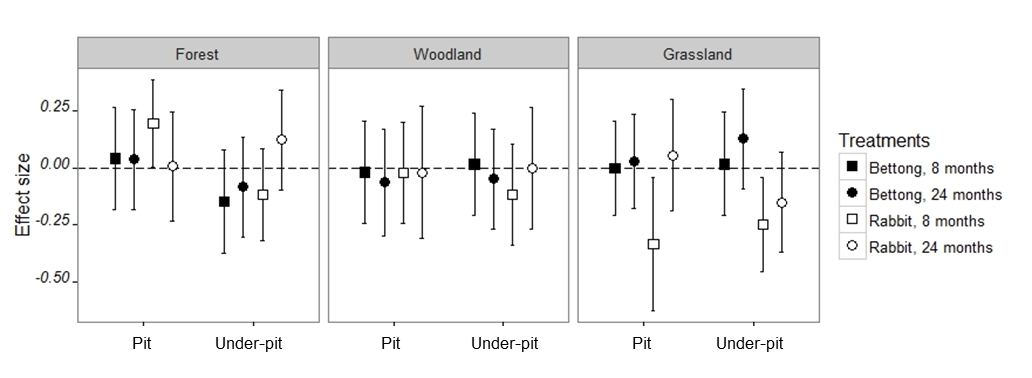


1. C:N ratio


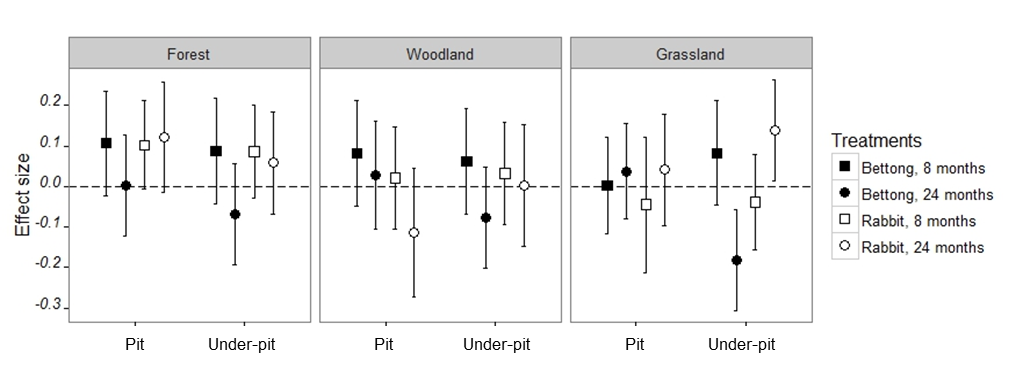


1. NH_4_+ µg/kg


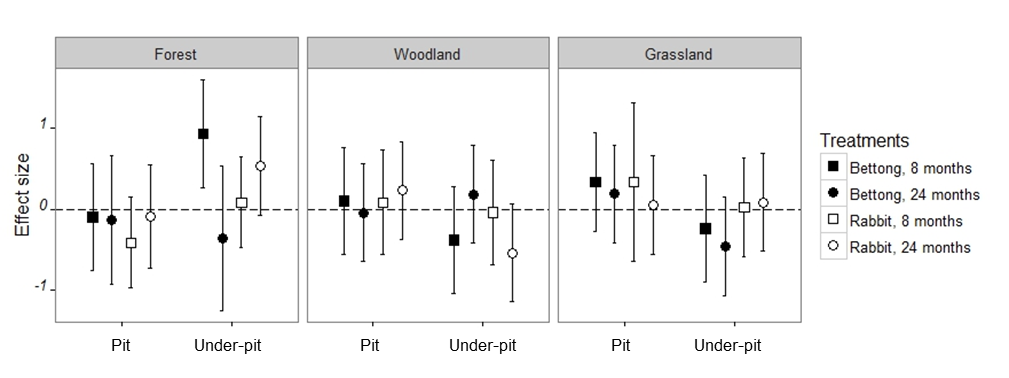


1. NO_3_- µg/kg


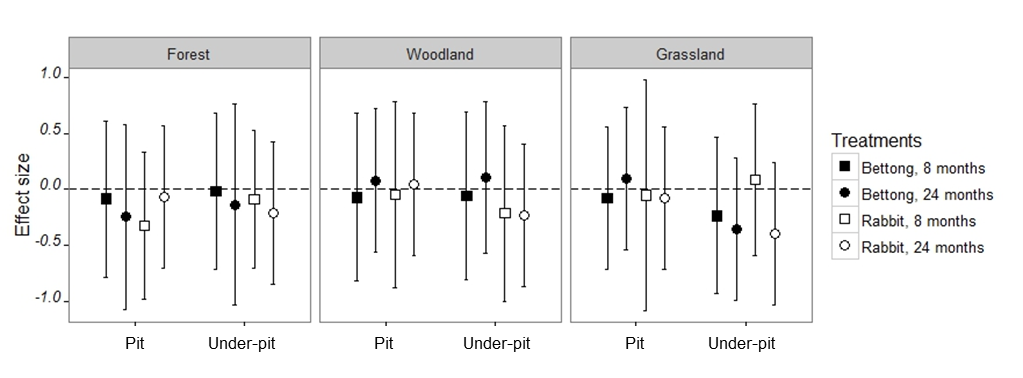


1. P µg/kg


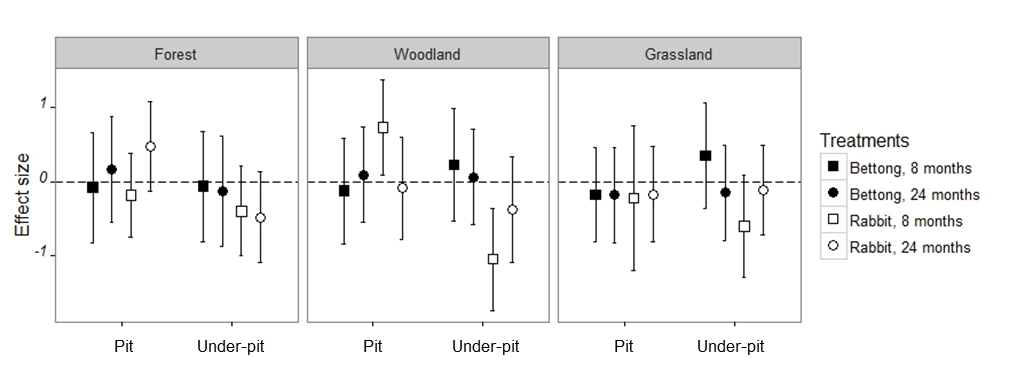


1. pH


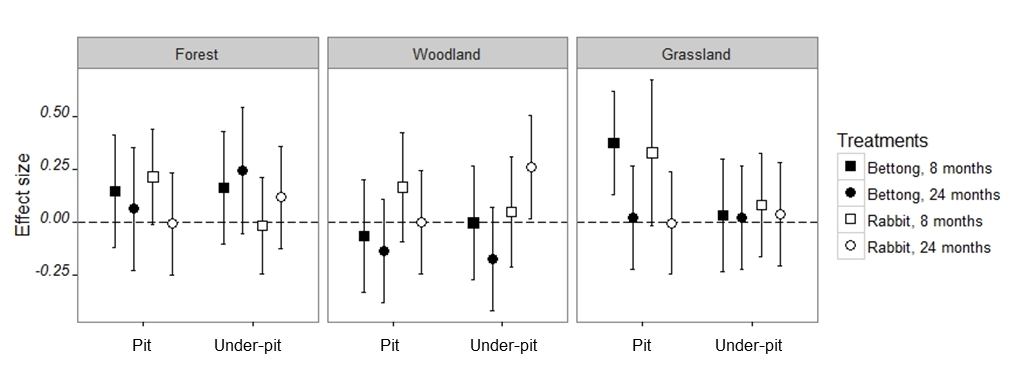


1. EC


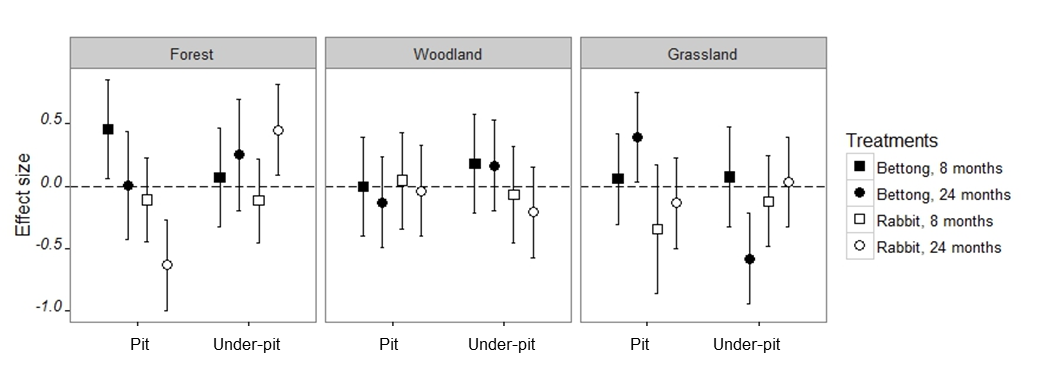


**Table S1 –** Summary of results of linear mixed models showing change in pit dimensions and volume over time.

| Response | Fixed effects | Estimate | Std. Error | df | t value | Pr(>\|t\|) |
| --- | --- | --- | --- | --- | --- | --- |
| Depth/radius | (Intercept) | 1.67 | 0.03 | 5.56 | 48.09 | **<0.001** |
|  | Age.months.8 | -1.11 | 0.03 | 442.96 | -31.80 | **<0.001** |
|  | Age.months.24 | -1.45 | 0.04 | 443.07 | -37.90 | **<0.001** |
|  | speciesrabbit | -1.07 | 0.03 | 442.96 | -30.56 | **<0.001** |
|  | Age.months.8:speciesrabbit | 0.79 | 0.05 | 442.97 | 15.97 | **<0.001** |
|  | Age.months.24:speciesrabbit | 1.07 | 0.06 | 443.09 | 19.35 | **<0.001** |
| Volume | (Intercept) | 31.95 | 3.53 | 3.54 | 9.04 | **<0.001** |
|  | Age.months.8 | -9.34 | 2.81 | 442.97 | -3.32 | **<0.001** |
|  | Age.months.24 | -21.86 | 3.08 | 443.03 | -7.09 | **<0.001** |
|  | speciesrabbit | 27.34 | 2.80 | 442.97 | 9.75 | **<0.001** |
|  | Age.months.8:speciesrabbit | -23.78 | 3.98 | 442.97 | -5.97 | **<0.001** |
|  | Age.months.24:speciesrabbit | -22.28 | 4.43 | 443.03 | -5.03 | **<0.001** |

**Table S2 –** Summary of results of Tukey’s post-hoc tests, based on predicted responses from the linear mixed models for pit dimensions and volume (see Table S1).

| Response | Age (months) | species | lsmean | SE | df | lower.CL | upper.CL | group |
| --- | --- | --- | --- | --- | --- | --- | --- | --- |
| Depth/radius | 24 | bettong | 0.22 | 0.04 | 8.32 | 0.09 | 0.35 | a |
|  | 24 | rabbit | 0.22 | 0.04 | 9.40 | 0.09 | 0.35 | a |
|  | 8 | rabbit | 0.29 | 0.03 | 5.82 | 0.15 | 0.42 | a |
|  | 8 | bettong | 0.56 | 0.03 | 5.76 | 0.42 | 0.70 | b |
|  | 0 | rabbit | 0.61 | 0.03 | 5.69 | 0.47 | 0.74 | b |
|  | 0 | bettong | 1.67 | 0.03 | 5.69 | 1.53 | 1.81 | c |
| Volume | 24 | bettong | 10.09 | 3.76 | 4.62 | -6.42 | 26.60 | a |
|  | 24 | rabbit | 15.15 | 3.84 | 5.01 | -0.94 | 31.24 | ab |
|  | 8 | bettong | 22.62 | 3.54 | 3.63 | 4.24 | 40.99 | bc |
|  | 8 | rabbit | 26.17 | 3.55 | 3.66 | 7.87 | 44.48 | cd |
|  | 0 | bettong | 31.95 | 3.53 | 3.60 | 13.50 | 50.41 | d |
|  | 0 | rabbit | 59.29 | 3.53 | 3.60 | 40.84 | 77.74 | e |

**R Code used for linear mixed models and effect size plots (See tables S1 and S2, Figure 2, Figure 5 and Figure S5a-h)**

**Pit dimensions - linear mixed models and pairwise significance testing**

library(lme4)

library(effects)

library(emmeans)data$Age.months.<-factor(data$Age.months.,levels=c("0","8","24"))

mod<-lmer(volume~Age.months.*species + (1|Vegtype),data=data)

effects<-allEffects(mod)

effects<-data.frame(effects$`Age.months.:species`)

effects$Age.months.<-factor(effects$Age.months.,levels=c("0","8","24"))

pw<-lsmeans(mod, pairwise ~ Age.months.*species)

ph<-CLD(pw,

alpha=0.05,

Letters=letters,

adjust="tukey")

ph<-data.frame(ph)

effects<-merge(ph, effects, by=c("Age.months.","species"))

effects$.group<-trimws(effects$.group)

dodge=position_dodge(width=0.5)

jpeg(file = "volume.jpeg", width = 3500, height = 3000, units = "px", res = 800)

plot=ggplot(data=effects, aes(y=fit,x=Age.months.,

ymin=lower,ymax=upper,group=species)) + theme_bw()

plot+

geom_line(aes(group=species),position=dodge,linetype=2)+

geom_text(aes(y=fit+10,x=Age.months.,label=.group),position=dodge)+

geom_errorbar(width=0.15,position = dodge)+

geom_point(size=3,shape=21,position=dodge,aes(fill=factor(species)))+

scale_fill_manual(values=c("bettong"="black","rabbit"="white"),labels=c("bettong"="Bettong","rabbit"="Rabbit"))+

ylab(expression(paste("Volume (",cm^3,")")))+

xlab("Age (months)")+

theme(axis.line = element_line(size=0),

panel.grid.major = element_blank(),

panel.grid.minor = element_blank(),

panel.background = element_blank(),

axis.text.y=element_text(angle=0, vjust=0.5,color="black"),

axis.text.x=element_text(angle=0,vjust=0.3,hjust=1,color="black"),

plot.title = element_text(hjust = 0.5),

strip.text.y = element_blank()) +

guides(shape = guide_legend(reverse=T),position="topright")+

labs(fill="Species",title="Volume of bettong and rabbit\npits over time")

dev.off()

#pit radius over time

mod<-lmer(depth.radius~Age.months.*species + (1|Vegtype),data=data)

effects<-allEffects(mod)

effects<-data.frame(effects$`Age.months.:species`)

effects$Age.months.<-factor(effects$Age.months.,levels=c("0","8","24"))

pw<-lsmeans(mod, pairwise ~ Age.months.*species)

ph<-CLD(pw,

alpha=0.05,

Letters=letters,

adjust="tukey")

ph<-data.frame(ph)

effects<-merge(ph, effects, by=c("Age.months.","species"))

effects$.group<-trimws(effects$.group)

jpeg(file = "pit.depth x radius.jpeg", width = 3500, height = 3000, units = "px", res = 800)

plot=ggplot(data=effects, aes(y=fit,x=Age.months.,

ymin=lower,ymax=upper,group=species)) + theme_bw()

plot+

geom_line(aes(group=species),position=dodge,linetype=2)+

geom_text(aes(y=fit+0.15,x=Age.months.,label=.group),position=dodge)+

geom_errorbar(width=0.15,position = dodge)+

geom_point(size=3,shape=21,position=dodge,aes(fill=factor(species)))+

scale_fill_manual(values=c("bettong"="black","rabbit"="white"),labels=c("bettong"="Bettong","rabbit"="Rabbit"))+

ylab("Pit depth/radius (cm)")+

xlab("Age (months)")+

theme(axis.line = element_line(size=0),

panel.grid.major = element_blank(),

panel.grid.minor = element_blank(),

panel.background = element_blank(),

axis.text.y=element_text(angle=0, vjust=0.5,color="black"),

axis.text.x=element_text(angle=0,vjust=0.3,hjust=1,color="black"),

plot.title = element_text(hjust = 0.5),

strip.text.y = element_blank()) +

guides(shape = guide_legend(reverse=T),position="topright")+

labs(fill="Species",title="Pit depth/radius over time")

dev.off()

pw<-lsmeans(mod, pairwise ~ Age.months.*species)

CLD(pw,

alpha=0.05,

Letters=letters,

adjust="tukey")

**Soil chemistry – linear mixed models and effect size plots**

library(lme4)

library(outliers)

library(ggplot2)

data$Age<-factor(data$Age,levels=c("8","24"))

data$Vegtype<-factor(data$Vegtype)

data$Animal<-factor(data$Animal)

data$Dig<-factor(data$Dig)

data$Treatment<-factor(data$Treatment)

new.data<-data

response<-log(new.data$ph+1)

response<-log(data.no.underdig$C.N.ratio+1)

mod<-lmer(response~Age*Vegtype*Animal*Dig/Treatment+(1|Site:dig.number),data=new.data,REML=F) #the model

mod<-lmer(NO3.N.mcg.kg.soil~Age*Vegtype*Animal*Dig/Treatment+(1|Site:dig.number),data=new.data,REML=F) #the model

mod<-lmer(response~Treatment+(1|Site:dig.number),data=data.no.underdig,REML=F) #the model

hist(residuals(mod))

qqnorm(residuals(mod))

plot(mod)

summary(mod)

CIs<-confint(mod)

CIs<-data.frame(CIs)

row.names(CIs)

CIs<-CIs[3:50,]

mod.dat<-data.frame(summary(mod)$coefficients)

mod.dat<-cbind(mod.dat,CIs)

mod.dat<-mod.dat[25:48,]

age<-rep(c("8 months","24 months"),12)

veg<-rep(c(rep("Forest",2),rep("Grassland",2),rep("Woodland",2)),4)

animal<-rep(c(rep("Bettong",6),rep("Rabbit",6)),2)

dig<-c(rep("Dig",12),rep("Underdig",12))

plotdat<-cbind(mod.dat,age,veg,animal,dig)

plotdat$veg.dig<-interaction(plotdat$dig,plotdat$veg)

plotdat$animal.dig<-interaction(plotdat$animal,plotdat$dig)

plotdat$age.animal<-interaction(plotdat$age,plotdat$animal)

plotdat$age<-factor(plotdat$age,levels=c("8 months","24 months"))

jpeg(filename = "ph plot.jpeg", width = 6000, height = 1800, units = "px",res = 800)

plot=ggplot(data=plotdat, aes(y=Estimate,x=dig,ymin=X2.5..,ymax=X97.5..,group=age.animal),xlab="",ylab="Effect size") + theme_bw()

plot+

geom_hline(yintercept=0,linetype="longdash")+

ggtitle("")+

geom_errorbar(width=0.15,position = position_dodge(width=0.7))+

geom_point(size=3,position=position_dodge(width=0.7),aes(shape=factor(age),fill=factor(animal)))+

scale_shape_manual(values=c("8 months"=22,"24 months"=21))+

scale_fill_manual(values=c("Bettong"="Yellow","Rabbit"="Purple"))+

facet_grid(. ~ veg)+

xlab("")+

ylab("Effect size")+

theme(axis.line = element_line(colour = "black"),

panel.grid.major = element_blank(),

panel.grid.minor = element_blank(),

panel.background = element_blank(),

plot.title = element_text(hjust = 0.5),

axis.text.y=element_text(angle=0, face="italic",vjust=0),

strip.text.y = element_blank())

dev.off()
